# Resistance of t(17;19)‐acute lymphoblastic leukemia cell lines to multiagents in induction therapy

**DOI:** 10.1002/cam4.2356

**Published:** 2019-07-15

**Authors:** Atsushi Watanabe, Takeshi Inukai, Keiko Kagami, Masako Abe, Masatoshi Takagi, Takashi Fukushima, Hiroko Fukushima, Toru Nanmoku, Kiminori Terui, Tatsuya Ito, Tsutomu Toki, Etsuro Ito, Junya Fujimura, Hiroaki Goto, Mikiya Endo, Thomas Look, Mark Kamps, Masayoshi Minegishi, Junko Takita, Toshiya Inaba, Hiroyuki Takahashi, Akira Ohara, Daisuke Harama, Tamao Shinohara, Shinpei Somazu, Hiroko Oshiro, Koshi Akahane, Kumiko Goi, Kanji Sugita

**Affiliations:** ^1^ Department of Pediatrics, School of Medicine University of Yamanashi Chuo Japan; ^2^ Department of Pediatrics and Developmental Biology, Graduate School of Medicine Tokyo Medical and Dental University Tokyo Japan; ^3^ Department of Child Health, Faculty of Medicine University of Tsukuba Tsukuba Japan; ^4^ Department of Clinical Laboratory University of Tsukuba Hospital Tsukuba Japan; ^5^ Department of Pediatrics Hirosaki University School of Medicine Hirosaki Japan; ^6^ Department of Pediatrics and Adolescent Medicine Juntendo University School of Medicine Tokyo Japan; ^7^ Hematology/Oncology & Regenerative Medicine Kanagawa Children's Medical Center; ^8^ Department of Pediatrics Iwate Medical University School of Medicine Morioka Japan; ^9^ Pediatric Oncology Dana‐Farber Cancer Institute Boston Massachusetts; ^10^ Department of Pathology University of California School of Medicine La Jolla California; ^11^ Tohoku Block Center Japanese Red Cross Society Sendai Japan; ^12^ Department of Pediatrics Kyoto University Graduate School of Medicine Kyoto Japan; ^13^ Department of Molecular Oncology, Research Institute for Radiation Biology and Medicine Hiroshima University Hiroshima Japan; ^14^ Tokyo Children's Cancer Study Group Tokyo Japan

**Keywords:** chemotherapy, hematalogical cancer, leukemia, pediatric cancer

## Abstract

t(17;19)(q21‐q22;p13), responsible for TCF3‐HLF fusion, is a rare translocation in childhood B‐cell precursor acute lymphoblastic leukemia(BCP‐ALL). t(1;19)(q23;p13), producing TCF3‐PBX1 fusion, is a common translocation in childhood BCP‐ALL. Prognosis of t(17;19)‐ALL is extremely poor, while that of t(1;19)‐ALL has recently improved dramatically in intensified chemotherapy. In this study, *TCF3‐HLF* mRNA was detectable at a high level during induction therapy in a newly diagnosed t(17;19)‐ALL case, while *TCF3‐PBX1* mRNA was undetectable at the end of induction therapy in most newly diagnosed t(1;19)‐ALL cases. Using 4 t(17;19)‐ALL and 16 t(1;19)‐ALL cell lines, drug response profiling was analyzed. t(17;19)‐ALL cell lines were found to be significantly more resistant to vincristine (VCR), daunorubicin (DNR), and prednisolone (Pred) than t(1;19)‐ALL cell lines. Sensitivities to three (Pred, VCR, and l‐asparaginase [l‐Asp]), four (Pred, VCR, l‐Asp, and DNR) and five (Pred, VCR, l‐Asp, DNR, and cyclophosphamide) agents, widely used in induction therapy, were significantly poorer for t(17;19)‐ALL cell lines than for t(1;19)‐ALL cell lines. Consistent with poor responses to VCR and DNR, gene and protein expression levels of P‐glycoprotein (P‐gp) were higher in t(17;19)‐ALL cell lines than in t(1;19)‐ALL cell lines. Inhibitors for P‐gp sensitized P‐gp‐positive t(17;19)‐ALL cell lines to VCR and DNR. Knockout of P‐gp by CRISPRCas9 overcame resistance to VCR and DNR in the P‐gp‐positive t(17;19)‐ALL cell line. A combination of cyclosporine A with DNR prolonged survival of NSG mice inoculated with P‐gp‐positive t(17;19)‐ALL cell line. These findings indicate involvement of P‐gp in resistance to VCR and DNR in Pgp positive t(17;19)‐ALL cell lines. In all four t(17;19)‐ALL cell lines, RAS pathway mutation was detected. Furthermore, among 16 t(1;19)‐ALL cell lines, multiagent resistance was usually observed in the cell lines with RAS pathway mutation in comparison to those without it, suggesting at least a partial involvement of RAS pathway mutation in multiagent resistance of t(17;19)‐ALL.

## 1 INTRODUCTION

For childhood B‐cell precursor acute lymphoblastic leukemia (BCP‐ALL), chromosomal translocation is strongly associated with therapeutic outcome.^1^, ^2^ t(17;19)(q21‐q22;p13) is a rare translocation and presents in less than 1% of childhood BCP‐ALL cases.^3^ Clinically, prognosis of t(17;19)‐ALL is extremely poor even in recently intensified chemotherapy.^4^ In t(17;19)‐ALL, the *TCF3* (*E2A*) gene on 19p13 fuses to the *HLF* gene on 17q21‐22 in‐frame.^5^, ^6^ TCF3‐HLF fusion acts as a transcription factor through the transactivation domains of TCF3 and a DNA‐binding and dimerization basic leucine zipper (bZIP) domain of HLF.^7^, ^8^ t(1;19)(q23;p13), which is another translocation involving the *TCF3* gene, is quite common translocation and presents in approximately 5% of childhood ALL cases.^9^ Prognosis of t(1;19)‐ALL has been dramatically improved in recently intensified chemotherapy.^10^, ^11^, ^12^ In t(1;19)‐ALL, the *TCF3* gene fuses in‐frame to the *PBX1* gene on 1q23.^13^, ^14^ TCF3‐PBX1 acts as the transcription factor through the transactivation domains of TCF3 and a homeobox DNA‐binding domain of PBX1.^13^, ^15^ Although both fusion transcription factors share the transactivation domains of TCF3, TCF3‐HLF and TCF3‐PBX1 regulate different downstream target genes by binding to different consensus nucleotide sequences through the bZIP domain of HLF and the homeobox domain of PBX1, respectively.^16^ Thus, distinctive prognosis between t(17;19)‐ALL and t(1;19)‐ALL may be attributed at least partially to differences in the transcriptional activities of TCF3‐HLF and TCF3‐PBX1.

Recent comprehensive genetic analyses of t(17;19)‐ALL and t(1;19)‐ALL using patient‐derived xenografts revealed significant differences between the molecular landscape of the two groups; deletions of *PAX5* and *VPREB1* and mutations of *TCF3* and RAS pathway genes such as *NRAS*, *KRAS*, and *PTPN11* were more frequently observed in t(17;19)‐ALL samples.^17^ These observations suggest an unconfirmed possibility that these additional genetic abnormalities may be involved in the poor therapeutic response of t(17;19)‐ALL in association with TCF3‐HLF. Consistent with the dismal outcome with chemotherapy, drug response profiling of patient‐derived t(17;19)‐ALL xenografts on human mesenchymal stroma cells using a coculture system revealed resistance to several standard chemotherapeutic agents such as vincristine (VCR) and cytarabine.^17^ However, t(17;19)‐ALL xenografts are significantly more sensitive to glucocorticoids than other high‐risk pre‐B and T‐ALL xenografts including t(1;19)‐ALL.^17^ Thus, further analyses are required to verify the drug response profiling of t(17;19)‐ALL in comparison with that of t(1;19)‐ALL.

In this study, using a panel of t(17;19)‐ALL and t(1;19)‐ALL cell lines, we analyzed drug response profiling in a simple liquid culture system. Prior to analyses of the cell lines, we prospectively examined the levels of minimal residual disease (MRD) during induction therapy in a newly diagnosed t(17;19)‐ALL case and t(1;19)‐ALL cases. We confirmed high level of MRD in the t(17;19)‐ALL case, indicating that (17;19)‐ALL shows resistance to chemotherapeutic agents in induction therapy. To verify this idea, we analyzed in vitro sensitivities to multiple chemotherapeutic agents of the t(17;19)‐ALL and t(1;19)‐ALL cell lines in induction therapy. We established that t(17;19)‐ALL cell lines were significantly more resistant to VCR, daunorubicin (DNR), and glucocorticoids than t(1;19)‐ALL cell lines. Furthermore, we confirmed comprehensive drug resistance to multiple agents in t(17;19)‐ALL cell lines in induction therapy.

## 2 MATERIALS AND METHODS

### 2.1 Minimal residual disease analyses

The patients were enrolled in Tokyo Children's Cancer Study Group (TCCSG) L04‐16 study.^18^ Minimal residual disease MRD was tested for by using bone marrow aspirates obtained at diagnosis and on days 15, 29, and 43. Real‐time polymerase chain reaction (PCR) analysis for *TCF3‐PBX1* was performed using sense (5′‐CCAGCCTCATGCACAACCA‐3) and antisense (5’‐ GGGCTCCTCGGATACTCAAAA‐3′) primers with probe (5′‐FAM‐CCCTCCCTGACCTGTCTCGGCC‐TAMRA‐3′), as previously described.^19^ The PCR mixture (50 µL total volume) consisted of sense primers and antisense primers (0.5 µmol/L each), TaqMan probes each at 100 nmol/L; dATP, dCTP, and dGTP, each at 200 µmol/L, and 400 µmol/L dUTP, 4 mmol/L MgCl_2_, 0.01 U Uracil DNA glycosylase per µL, 0.025 U of AmpliTaq Gold per microliter, and 1 × TaqMan PCR buffer (Thermo Fisher Scientific, Waltham, MA). Amplification and detection were performed using an ABI 7900 sequence detection system (Applied Biosystems, Foster City, CA). The data were analyzed by Sequence Detector version 1.63 software (Applied Biosystems). The final results were normalized by the amount of internal control *GAPDH* (Thermo Fisher Scientific). Real‐time PCR analysis for *TCF3/HLF* was performed using sense (5′‐GCCTCATGCACAACCACGCG‐3) and antisense (5′‐CCCGGATGGCGATCTGGTTC‐3′) primers with a SYBR Green PCR Master Mix (Applied Biosystems). As an internal control for *TCF3/HLF* quantification, *ACTB* was quantified using sense (5'‐ACCTTCTACAATGAGCTGCGT‐3') and antisense (5'‐GTACATGGCTGGGGTGTTGA‐3') primers.

### 2.2 Leukemia cell lines

Four t(17;19)‐ALL cell lines (UOC‐B1, HALO1, YCUB2, and Endo‐kun) and 16 t(1;19)‐ALL cell lines (KOPN‐K, ‐34, ‐36, ‐54, ‐60, ‐63, YAMN‐90, ‐92, YCUB6, YCUB8, Kasumi2, SCMC‐L1, THP4, 697, RCH, and PreALP) were used in this study.^19^ As B‐precursor ALL cell lines, seven *MLL*‐rearranged (*MLL*+) ALL cell lines (KOPN‐1, KOCL‐33, ‐44, ‐45, ‐50, ‐58, and ‐69), six Philadelphia chromosome (Ph)‐positive ALL cell lines (KOPN‐30bi, ‐57bi, ‐66bi, ‐72bi, YAMN‐73, and SU‐Ph2), and eight other ALL cell lines (KOPN‐32, ‐35, ‐41, ‐62, ‐70, ‐79, Reh, and Nalm6) were used (Table S1).^20^ All cell lines were maintained in RPMI1640 medium supplemented with 10% fetal calf serum (FCS) in a humidified atmosphere of 5% CO_2_ at 37°C.

### 2.3 AlamarBlue cell viability assay

To determine IC_50_s of DNR, VCR, prednisolone (Pred), dexamethasone (Dex), l‐asparaginase (l‐Asp), cyclophosphamide (CPM), and selumetinib, an alamarBlue assay was performed.^20^ The sources of the drugs are shown in Table S2. For CPM sensitivity, mafosfamide (MAF), an active analog of CPM, was used. Cells (1‐4 × 10^5^) were plated onto a 96‐well flat‐bottom plate in triplicate in the absence or presence of seven concentrations of each drug. The cells were cultured for 44 hours to determine the DNR, VCR, and CPM sensitivities and for 68 hours to determine Pred, Dex, l‐Asp, and selumetinib sensitivities, and, then, 20 µL of alamarBlue was added. After a 6‐hours additional incubation with alamarBlue, absorbance at 570 nm was monitored by a microplate spectrophotometer using 600 nm as a reference wavelength. Cell survival was calculated by expressing the ratio of the optical density of the treated wells to that of the untreated wells as a percentage. The concentration of agent required to reduce the viability of the treated cells to 50% of the untreated cells was calculated, and the median of three independent assays was determined as IC_50_. The median of the IC_50_s measured by three independent assays was determined.

### 2.4 Flow cytometric analysis

To detect apoptotic events, cells were cultured in the absence or presence of DNR or VCR in combination with or without verapamil, cyclosporine A (CyA), or nilotinib for 24 hours, and stained with a fluorescein isothiocyanate‐conjugated Annexin‐V (BioLegend, San Diego, CA) and actinomycin‐D (Sigma‐Aldrich, St Louis, MO). Cell surface expression of P‐gp was analyzed using a phycoerythrin‐conjugated anti‐P‐gp antibody. For the functional assay of P‐gp‐mediated efflux of calcein‐AM (CAM), HALO1 cells were incubated with 0.25 mmol/L of CAM for 10 minutes at 37°C in the absence or presence of velapamil, CyA, or nilotinib. The stained cells were analyzed by flow cytometry (FACSCalibur, BD Biosciences, San Jose, CA).

### 2.5 Combined sensitivities to multiple agents

The combined sensitivities of 4 t(17;19)‐ALL and 16 t(1;19)‐ALL cell lines to three (Pred, VCR, and l‐Asp), four (Pred, VCR, l‐Asp, and DNR), and five (Pred, VCR, l‐Asp, DNR, and CPM) drugs were analyzed according to previous reports.^21^, ^22^ Twenty cell lines were classified into three equal‐sized groups as either sensitive (33% lowest IC_50_ values; seven cell lines), intermediately sensitive (33% intermediate IC_50_ values; six cell lines), or resistant (33% highest IC_50_ values; seven cell lines) to each drug. A sensitive result was rated as 1, an intermediate result as 2, and a resistant result as 3, and the total score was calculated by adding these counts.

### 2.6 Real‐time RT‐PCR analysis

Total RNA was extracted using the Trizol reagent (Invitrogen, Carlsbad, CA), reverse transcription was performed using a random hexamer (Amersham Bioscience, Buckinghamshire, United Kingdom) by Superscript II reverse transcriptase (Invitrogen), and then incubation with RNase (Invitrogen). For quantitative real‐time PCR, triplicated samples containing cDNA with TaqMan Universal PCR Master Mix (Applied Biosystems) and Gene Expression Product listed in Table 1 were amplified following manufacturer's protocol using UOCB1 as a control. As an internal control for relative gene expression, quantitative real‐time PCR for *ACTB* was performed.

**Table 1:** TaqMan probe used in the study

Genes	TaqMan probes
*P‐gp*(*MDR1*, *ABCB1*)	Hs00184500_m1
*BCRP1*(*ABCP*, *ABCG2*)	Hs01053790_m1
*LRP*(*MVP*)	Hs00245438_m1
*MRP1*(*ABCC1*)	Hs01561502_m1
*MRP2*(*ABCC2*)	Hs00166123_m1
*MRP3*(*ABCC3*)	Hs00978473_m1
*MRP4*(*ABCC4*)	Hs00988717_m1
*MRP5*(*ABCC5*)	Hs00981087_m1
*MRP6*(*ABCC6*)	Hs00184566_m1
βactin(ACTB)	Hs01060665_g1

### 2.7 In vivo analysis of drug sensitivity

Six‐week‐old female NSG (NOD.Cg‐PrkdcscidIl2rgtm1Wjl/SzJ) mice were purchased from Jackson Laboratory (Bar Harbor, ME, USA). The experiment was performed in a specific pathogen‐free unit after approval of protocols for animal care and experiment by the Tokyo Medical and Dental University animal care and use committee (approved No. A2017113). HALO1 cells (1 x 10^4^) were injected into the tail vein to establish xenografts. One day after injection of HALO1 cells, each of the five mice were treated with 0.5 mg/kg of DNR alone or 0.5 mg/kg of DNR in combination with 50 mg/kg of CyA for five consecutive days. DNR and CyA were further diluted with phosphate‐ buffered saline (PBS) and intraperitoneally injected into the mice. CyA was injected one hour before administration of DNR. The control group of five mice was administered PBS only.

### 2.8 Knockout of P‐glycoprotein with CRISPR‐Cas9 system

To knockout P‐glycoprotein (P‐gp; ABCB1) expression with the CRISPR/Cas9 system, we selected 5′‐TTTGGCTGCCATCATCCATGG‐3′, which showed the highest off‐target hit score in the CRISPR design tool (CRISPR DESIGN, http://crispr.mit.edu), and the synthesized oligomers were cloned into CRISPR/Cas9 vectors (CRISPR CD4 Nuclease Vector, Thermo Fisher Scientific, Waltham, MA). Three days after electroporation of the ABCB1‐targeting CRISPR/Cas9 vector into HALO1 cells using the Neon electroporation transfection system (Thermo Fisher Scientific), CD4‐positive cells were selected using CD4 microbeads (Miltenyi Biotec, Auburn, CA) and expanded for further analyses.^20^

### 2.9 Target deep sequencing of RAS pathway genes

Target deep sequencing of RAS pathway genes including the *PTPN11*, *NRAS*, *KRAS*, and *NF1* were analyzed using SureDesign software (Agilent Technologies, Santa Clara, CA). Libraries were prepared using the HaloPlex Target Enrichment System (Agilent Technologies), followed by paired‐end sequencing on a MiSeq instrument (Illumina, San Diego, CA). Bioinformatic analysis was performed using the SureCall software (Agilent Technologies). Common germline polymorphisms reported in public databases were excluded and nonsense, frameshift, splice site, nonsynonymous variants were considered as mutations. Minimal allele frequency for mutation calling was set at 0.3.

## 3 RESULTS

### 3.1 MRD analysis of a newly diagnosed t(17;19)‐ALL case

**Figure 1:**
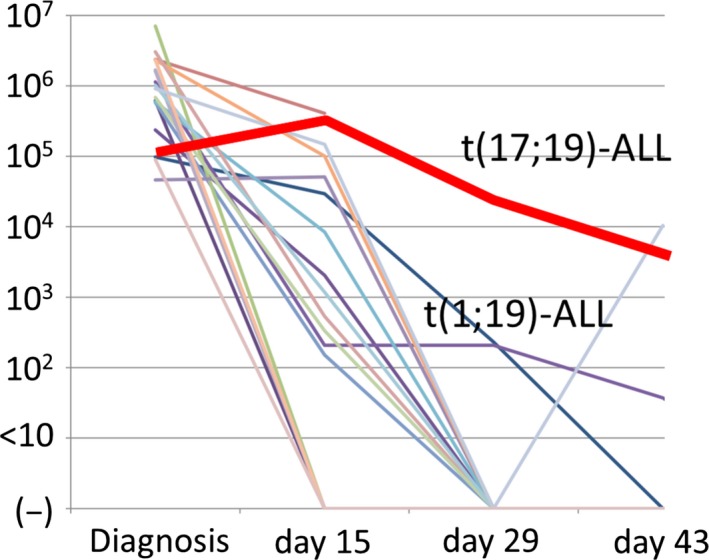
Prospective minimal residual disease (MRD) analysis in a newly diagnosed t(17;19)‐ALL case and t(1;19)‐ALL cases by real‐time RT‐PCR targeting of *TCF3‐HLF *and *TCF3‐PBX1 *chimeric mRNA, respectively. Levels of *TCF3‐HLF *and *TCF3‐PBX1 *chimeric mRNA in bone marrow aspirates were monitored at diagnosis and on days 15, 29, and 43 in induction therapy in a t(17;19)‐ALL case (red bold line) and 16 t(1;19)‐ALL cases, respectively, treated with the TCCSG L0416 high risk protocol

We prospectively evaluated MRD levels in a t(17;19)‐ALL case, treated with a high risk (HR) regimen in the TCCSG L04‐16 study,^18^ using real‐time RT‐PCR targeting of *TCF3‐HLF* (Figure 1). Higher levels of *TCF3‐HLF* chimeric mRNA were continuously detected during intensified induction therapy consisting of Pred, VCR, DNR, l‐Asp, and CPM. Bone marrow relapse was confirmed in the patient at the end of early intensification therapy. The patient received haploidentical transplantation from his mother, but regrowth of leukemic blasts was confirmed in the bone marrow on day 36. He was treated with donor lymphocyte infusions and obtained immediate remission. His bone marrow remained in complete remission for over 3 years.^23^ We also performed prospective evaluation of MRD levels in 16 consecutive cases of t(1;19)‐ALL, treated with an identical regimen, by real‐time RT‐PCR targeting of *TCF3‐PBX1*. *TCF3‐PBX1* chimeric mRNA constantly decreased during induction therapy and became undetectable on day 43 except in two cases (12.5%).

### 3.2 Chemoresistance of t(17;19)‐ALL cell lines

**Figure 2:**
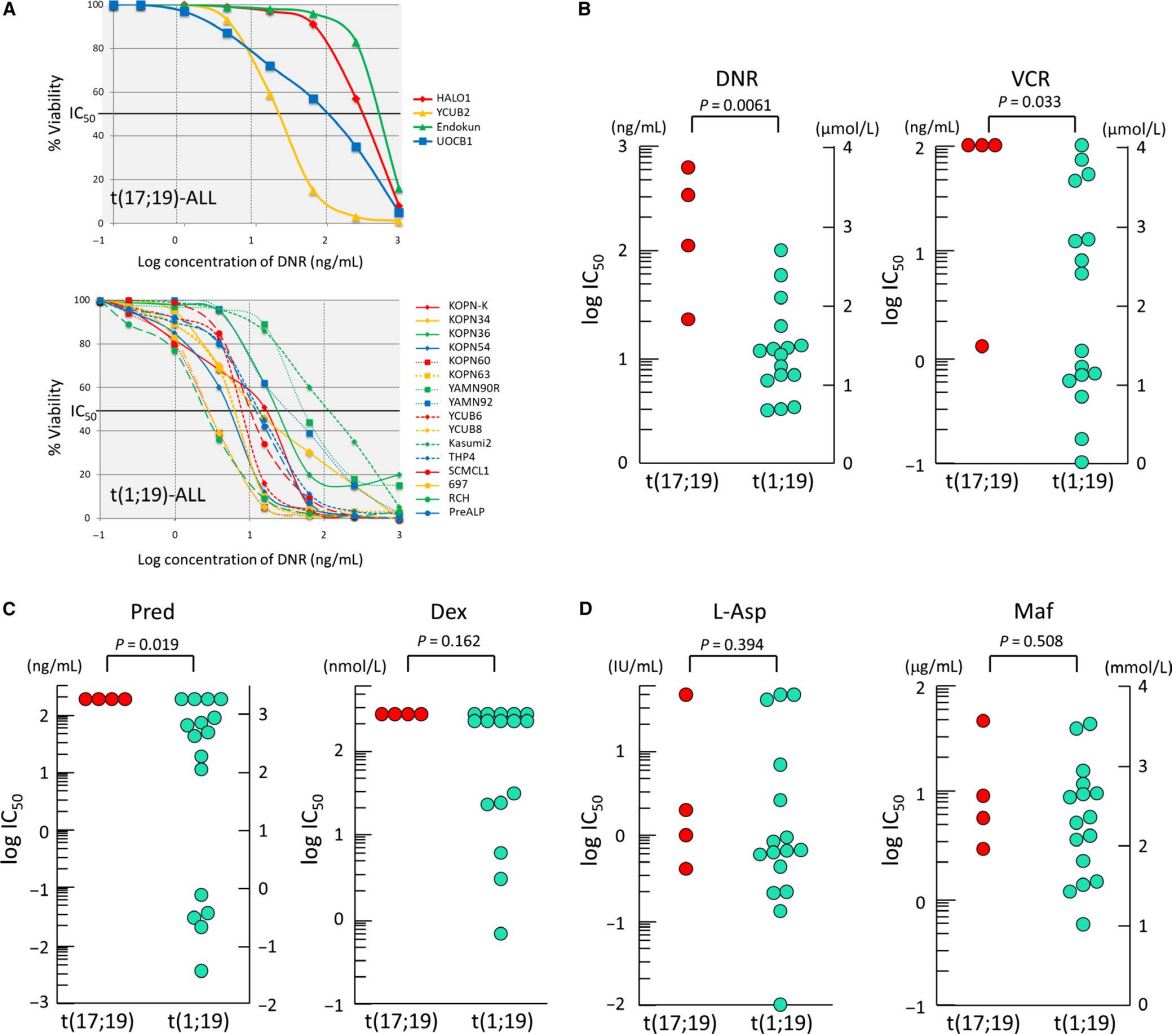
Sensitivities to agents in induction therapy in the t(17;19)‐ALL and t(1;19)‐ALL cell lines. (A) Dose response curves to daunorubicin (DNR) in t(17;19)‐ALL (top panel) and t(1;19)‐ALL (bottom panel) cell lines. Horizontal and vertical axis respectively indicates log concentration of DNR and cell viability determined by alamarBlue cell viability assay. (B, C, and D) Comparison of IC50s for DNR (B), VCR (B), Pred (C), Dex (C), L‐Asp (D), and Maf (D) between four t(17;19)‐ALL cell lines and 16 t(1;19)‐ALL cell lines. Pvalues in Mann‐Whitney test are shown

Although only a single case was observed, MRD analysis demonstrated resistance to induction therapy in the t(17;19)‐ALL case in comparison with the t(1;19)‐ALL cases. To verify resistance of t(17;19)‐ALL to induction therapy in vitro, we analyzed the sensitivities of four t(17;19)‐ALL cell lines, as well as 16 t(1;19)‐ALL cell lines, to six agents (Dex, Pred, VCR, DNR, l‐Asp, and CPM) used in the induction therapy of the HR regimen of the TCCSG L04‐16 study. We determined the IC_50_ of each agent based on the dose‐response curve in an alamarBlue cell viability assay (Table 2). As representatively shown in Figure 2A, t(17;19)‐ALL cell lines are significantly more resistant to DNR than t(1;19)‐ALL cell lines. IC_50_ of DNR in t(17;19)‐ALL cell lines (median: 300 ng/mL) is significantly higher than that of t(1;19)‐ALL cell lines (median: 15 ng/mL) (*P* = 0.006 in Mann‐Whitney test) (Figure 2B). IC_50_s of VCR (*P* = 0.033) (Figure 2B) and Pred (*P* = 0.019) (Figure 2C) in t(17;19)‐ALL cell lines are also significantly higher than those in t(1;19)‐ALL cell lines. Furthermore, although not statistically significant, IC_50_ of Dex in t(17;19)‐ALL cell lines tended to be higher than that in t(1;19)‐ALL cell lines (Figure 2C). Regarding sensitivities to l‐Asp and CPM, although median IC_50_s are almost identical between the two groups of cell lines, highly sensitive cell lines are relatively uncommon in t(17;19)‐ALL cell lines in comparison with t(1;19)‐ALL cell lines (Figure 2D).

**Table 2:** IC_50_ of t(17;19)‐ALL and t(1;19)‐ALL cell lines

Cell line	Daunorubicin		Vincristine		Prednisolone		Dexamethasone		l‐asparaginase		Mafosfamide	
ng/mL		ng/mL		nmol/L		nmol/L		IU/mL		μg/ml	
Median	Range	Median	Range	Median	Range	Median	Range	Median	Range	Median	Range
t(17;19)												
HALO1	303	122‐338	>100	>100	>150	>150	>250	>250	0.98	0.47‐1	0.9	0.39‐1.21
YCUB2	21	11‐27	1.4	1.1‐1.4	>150	>150	>250	>250	0.34	0.24‐0.74	0.43	0.3‐0.8
Endokun	512	355‐586	>100	60‐>100	>150	>150	>250	>250	>40	>40	3.9	3.4‐5.7
UOCB1	122	96‐294	>100	>100	>150	>150	>250	>250	1.94	0.9‐7.6	0.24	0.23‐0.32
t(1;19)												
KOPN‐K	16.1	9.8‐37	12.5	7.3‐32	0.003	0.003	0.48	0.25‐0.9	0.01	0.003‐0.08	1.17	0.77‐2.7
KOPN34	11.4	9.5‐26	43	35‐62	64	55‐79	>250	>250	33	9.6‐>40	0.95	0.71‐1.13
KOPN36	11.5	8.9‐22	67	50‐>100	10.9	0.19‐36	4.9	0.9‐5	0.61	0.43‐2.9	3.58	1.75‐7.8
KOPN54	5.3	5.2‐9.7	7.2	0.75‐9.7	0.018	0.009‐0.02	2.6	2.6‐3	0.85	0.59‐0.99	0.2	0.12‐0.27
KOPN60	2.7	1.4‐2.7	0.1	0.05‐0.27	0.072	0.044‐0.103	21	18‐90	0.36	0.21‐0.5	0.13	0.07‐0.26
KOPN63	6	1.6‐6	0.52	0.43‐0.85	>150	>150	>250	>250	0.56	0.44‐1.45	0.44	0.23‐0.8
YAMN90R	52	19‐82	37	18‐69	19	3.8‐>150	>250	58‐>250	>40	>40	1.36	1.07‐1.53
YAMN92	32.6	30‐119	13.5	6.5‐31	>150	>150	>250	>250	5.7	1.6‐6.2	3.2	1.16‐4.04
YCUB6	7.8	2.7‐8.8	0.76	0.76‐1.7	84	58‐90	>250	>250	0.62	0.003‐3.8	0.3	0.3‐0.5
YCUB8	6	4.6‐11	1.2	1.2‐2.4	43	36‐>150	>250	239‐>250	0.14	0.13‐0.3	0.11	0.03‐0.18
Kasumi2	106	51‐160	>100	>100	>150	>150	>250	>250	0.91	0.37‐1.1	0.804	0.33‐0.83
THP4	12.8	12.3‐28.1	0.36	0.27‐0.78	>150	>150	>250	>250	0.52	0.37‐0.56	0.32	0.18‐0.6
SCMC‐L1	10.1	3.4‐44.6	5.2	1.5‐43.3	36	31‐>150	>250	>250	>40	>40	0.92	0.28‐1.41
697	2.8	2.2‐4.9	0.6	0.51‐0.6	0.026	0.024‐0.054	26	16‐82	0.2	0.11‐0.21	0.048	0.039‐0.05
RCH	2.5	1.8‐7.9	0.62	0.28‐1.46	0.033	0.0083‐0.04	21.9	21.5‐33	2.3	1.2‐2.5	0.4	0.27‐0.4
PreALP	11.6	1.6‐21.8	0.15	0.02‐0.41	53	43‐61	>250	>250	0.2	0.13‐0.3	0.15	0.1‐0.18

We next verified an induction of apoptosis by DNR and VCR by determining cell viabilities using flow cytometry. We analyzed four t(17;19)‐ALL and seven representative t(1;19)‐ALL cell lines. When treated with 50 ng/mL of DNR, cell viabilities in two t(17;19)‐ALL cell lines (UOCB1 and HALO1) and two t(1;19)‐ALL cell lines (KOPN60 and 697) were 80%, 83%, 6%, and 4%, respectively (Figure S1A). A similar pattern was observed when treated with 50 ng/mL of VCR (Figure S1B). Cell viabilities in the DNR‐treated t(17;19)‐ALL cell lines (median: 68%) were relatively higher (*P* = 0.089 in Mann‐Whitney test) than those in the DNR‐treated t(1;19)‐ALL cell lines (22%) (Figure S1C). Similarly, cell viabilities in the VCR‐treated t(17;19)‐ALL cell lines (median: 85%) were significantly higher (*P* = 0.038) than those in the VCR‐treated t(1;19)‐ALL cell lines (55%) (Figure S1C).

### 3.3 Multiagent resistance in t(17;19)‐ALL cell lines

**Figure 3:**
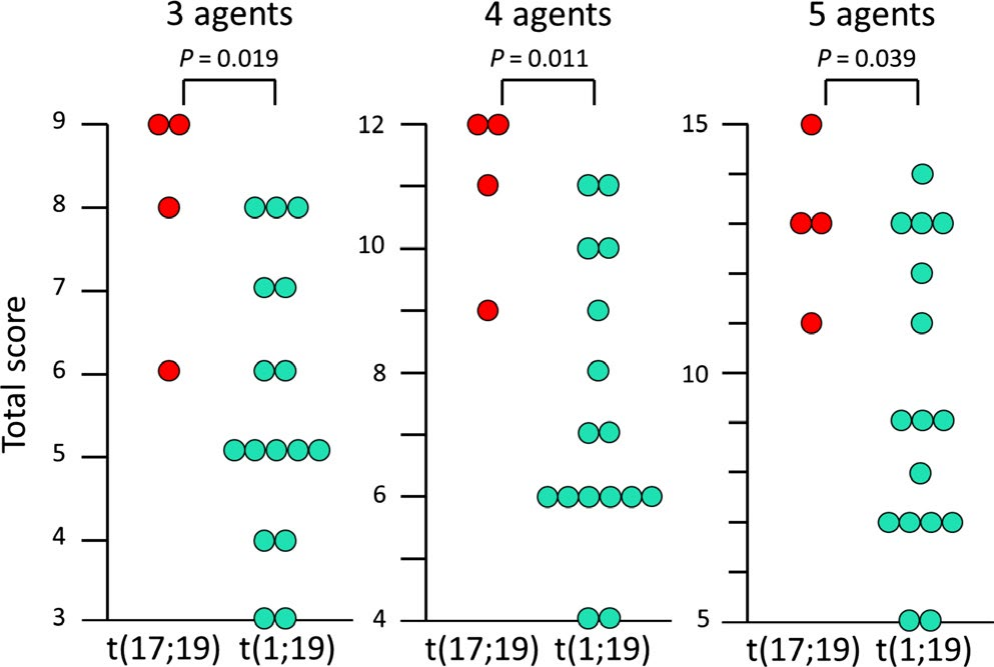
Multi‐agent resistance in t(17;19)‐ALL cell lines. Total score of sensitivities to three (Pred, VCR, and LAsp), four (Pred, VCR, L‐Asp, and DNR), and five (Pred, VCR, L‐Asp, DNR, and MAF) agents were compared between t(17;19)‐ALL and t(1;19)‐ALL cell lines. P‐values in Mann‐Whitney test are indicated

To comprehensively evaluate sensitivity of t(17;19)‐ALL cell lines to multiple agents used in induction therapy, we analyzed the combined sensitivities to three (Pred, VCR, and l‐Asp), four (Pred, VCR, l‐Asp, and DNR) and five (Pred, VCR, l‐Asp, DNR, and CPM) agents (Table 3) according to previous reports.^21^, ^22^ Total scores of sensitivities in t(17;19)‐ALL cell lines were significantly higher than those in t(1;19)‐ALL cell lines (*P* = 0.019 for three agents, *P* = 0.011 for four agents, and *P* = 0.039 for five agents; Figure 3), indicating that t(17;19)‐ALL cell lines were far more resistant to the multiple agents commonly used in induction therapy than t(1;19)‐ALL cell lines.

**Table 3:** Comprehensive analysis of multiagent resistance

Cell line	Prednisolone		Vincristine		l‐asparaginase		Three agents	Daunorubicin		Four agents	Mafosfamide		Five agents
	nmol/L		ng/mL		IU/mL			ng/mL			μg/mL		
	IC_50_	Score	IC_50_	Score	IC_50_	Score	Total score	IC_50_	Score	Total score	IC_50_	Score	Total score
t(17;19)													
HALO1	>150	3	>100	3	0.98	2	8	303	3	11	0.9	2	13
YCUB2	>150	3	1.4	2	0.34	1	6	21	3	9	0.43	2	11
Endokun	>150	3	>100	3	>40	3	9	512	3	12	3.9	3	15
UOCB‐1	>150	3	>100	3	1.94	3	9	122	3	12	0.24	1	13
t(1;19)													
KOPN‐K	0.003	1	12.5	2	0.01	1	4	16.1	2	6	1.17	3	9
KOPN34	64	2	43	3	33	3	8	11.4	2	10	0.95	3	13
KOPN36	10.9	1	67	3	0.61	2	6	11.5	2	8	3.58	3	11
KOPN54	0.018	1	7.2	2	0.85	2	5	5.3	1	6	0.2	1	7
KOPN60	0.072	1	0.1	1	0.36	1	3	2.7	1	4	0.13	1	5
KOPN63	>150	3	0.52	1	0.56	2	6	6	1	7	0.44	2	9
YAMN90R	19	1	37	3	>40	3	7	52	3	10	1.36	3	13
YAMN92	>150	3	13.5	2	5.7	3	8	32.6	3	11	3.2	3	14
YCUB6	84	2	0.76	1	0.62	2	5	7.8	1	6	0.3	1	7
YCUB8	43	2	1.2	2	0.14	1	5	6	1	6	0.11	1	7
Kasumi2	>150	3	>100	3	0.91	2	8	106	3	11	0.804	2	13
THP4	>150	3	0.36	1	0.52	1	5	12.8	2	7	0.32	2	9
SCMC‐L1	36	2	5.2	2	>40	3	7	10.1	2	9	0.92	3	12
697	0.026	1	0.6	1	0.2	1	3	2.8	1	4	0.048	1	5
RCH	0.033	1	0.62	1	2.3	3	5	2.5	1	6	0.4	2	8
PreALP	53	2	0.15	1	0.2	1	4	11.6	2	6	0.15	1	7

### 3.4 Expression of ABC transporters in t(17;19)‐ALL cell lines

**Figure 4:**
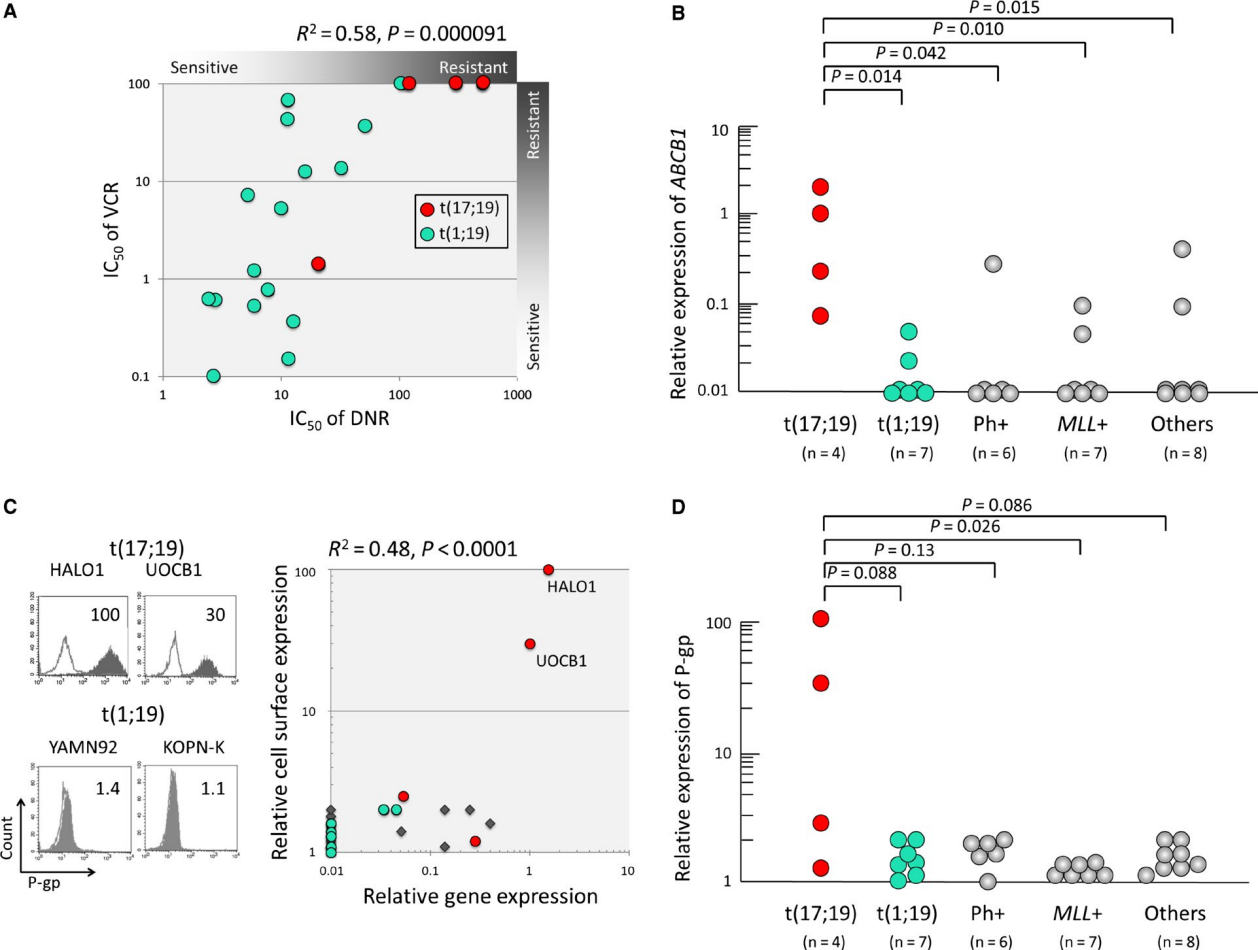
P‐glycoprotein expression in t(17;19)‐ALL cell lines. (A) Correlation between IC50 of VCR and that of DNR in t(17;19)‐ALL and t(1;19)‐ALL cell lines. (B) Gene expression level of *ABCB1 *in BCP‐ALL cell lines. *ABCB1 *gene expression was quantified by real time RT‐PCR using beta‐actin expression as an internal control. P‐values in Mann Whitney‐test are indicated on the top. (C) The cell surface expression of Pglycoprotein (P‐gp) on t(17;19)‐ALL cell lines. In the left pane, representative histograms of P‐gp expression are shown with relative fluorescence index (RFI). Black‐filled histograms represent anti‐P‐gp antibody staining and black‐line histograms represent isotype controls for staining. In the right panel, correlation between RFI of P‐gp expression (vertical axis) and relative expression level of *ABCB1 *(horizontal axis) in BCP‐ALL cell lines is presented. Red and green circles represent t(17;19)‐ALL cell lines and t(1;19)‐ALL cell lines, respectively, and squares represent other BCP‐ALL cell lines. (D) The cell surface expression of P‐gp in BCP‐ALL cell lines. P‐values in Mann Whitney‐test are indicated on the top

The IC_50_s of DNR in t(17;19)‐ALL and t(1;19)‐ALL cell lines were closely correlated with that of VCR (R^2^ = 0.58, *P* = 000,091) (Figure 4A). Since both DNR and VCR are sensitive to ABC transporters,^24^, ^25^ we quantified the gene expression level of the ABC‐transporter family members in BCP‐ALL cell lines. We performed real‐time RT‐PCR analyses of *LRP*, *P‐gp* (*MRD1*, *ABCB1*), *MRP1* (*ABCC1*), *MRP2* (*ABCC2*), *MRP3* (*ABCC3*), *MRP4* (*ABCC4*), *MRP5* (*ABCC5*), *MRP6* (*ABCC6*), and *BCRP* (*ABCG2*). Among nine genes, *MRP3*, *MRP5*, and *MRP6* were undetectable in BCP‐ALL cell lines. Gene expression levels of *ABCB1* were significantly higher in t(17;19)‐ALL cell lines than in other BCP‐ALL cell lines including the t(1;19)‐ALL cell lines (Figure 4B). In contrast, gene expression levels of *MRP1* were significantly lower in t(17;19)‐ALL cell lines than in other BCP‐ALL cell lines, although they were almost equal between the t(17;19)‐ALL cell lines and t(1;19)‐ALL cell lines (Figure S2). No significant differences were observed in *LRP*, *MRP2*, *MRP4*, and *BCRP* expression levels between t(17;19)‐ALL cell lines and other BCP‐ALL cell lines. Since *ABCB1* gene expression levels were significantly higher in t(17;19)‐ALL cell lines, we next analyzed the cell surface expression of P‐gp using flow cytometry. Among four t(17;19)‐ALL cell lines, P‐gp was clearly detectable in HALO1 and UOCB1 and marginally detectable in Endo‐kun, but almost undetectable in YCUB2 (Figure 4C). The cell surface expression level of P‐gp was correlated with the *ABCB1* gene expression level (*R* ^2^ = 0.48, *P* < 0.0001) in 32 BCP‐ALL cell lines. Cell surface expression level of P‐gp was relatively higher in t(17;19) cell lines than in t(1;19)‐ALL cell lines (*P* = 0.088 in Mann‐Whitney test) (Figure 4D). Further flow cytometry analysis of P‐gp revealed that all 16 t(1;19)‐ALL cell lines were negative or almost undetectable (data not shown).

### 3.5 Involvement of P‐gp in resistance to DNR and VCR of t(17;19)‐ALL cell lines

**Figure 5:**
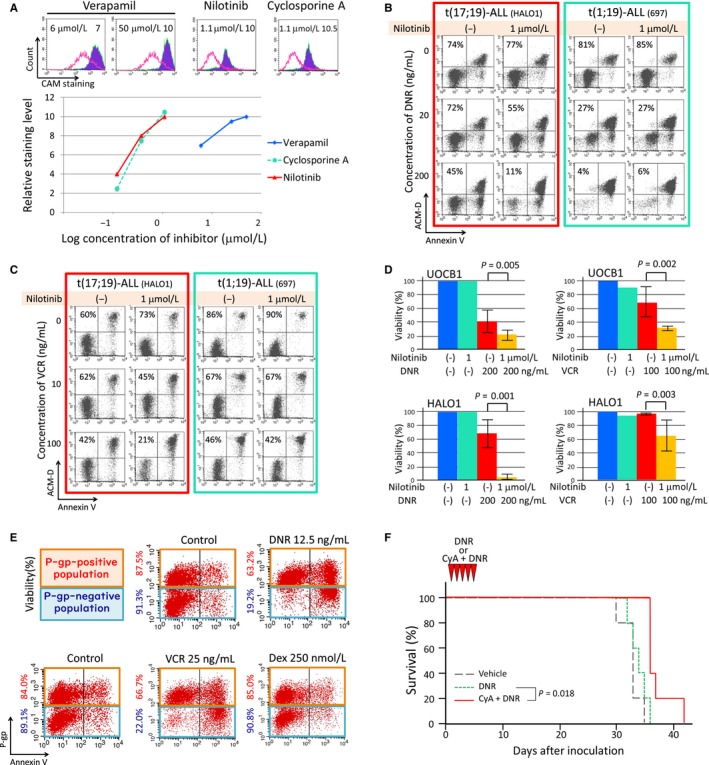
Involvement of P‐gp in DNR and VCR‐resistance in P‐gp‐positive t(17;19)‐ALL cell lines. (A) Increased efflux activity in HALO1, a P‐gp‐positive t(17;19)‐ALL cell line. HALO1 cells stained with calcein AM (CAM) were incubated in the absence or presence of verapamil, cyclosporine A, or nilotinib for 30 min at 37°C and, then, analyzed by flow cytometry. In the top panel, red‐line histograms and blue‐filled histograms represent CAM staining in the absence and presence of an inhibitor, respectively. Relative staining level is shown in each panel. In the bottom panel, the vertical axis indicates relative CAM staining level and the horizontal axis indicates log concentration of inhibitors. (B and C) Induction of apoptotic cell death by DNR (B) or VCR (C) in a combination with nilotinib. HALO1 (left panel) and 697 (right panel), a P‐gp‐negative t(1;19)‐ALL cell line, were cultured in the absence or presence of DNR (20 and 200 ng/mL) or VCR (10 and 100 ng/mL) overnight and then analyzed for Annexin V‐binding (horizontal axis) and actinomycin‐D (ACMD)‐ staining (vertical axis) by flow cytometry. Percentages of living cells (lower left) are pointed out in each panel. (D) Sensitivities to DNR (left panel) and VCR (right panel) in combination with nilotinib in HALO1 and UOCB1, P‐gp‐positive t(17;19)‐ALL cell lines. The vertical axis indicates median cell viability in triplicated alamarBlue cell viability assay. Error bar indicates standard deviation. P‐values in student T‐test between viability of cells treated with DNR or VCR alone and that of cells treated with DNR or VCR in combination with nilotinib. (E) Effect of P‐gp knockout on VCR, DNR, or Dex sensitivities in HALO1 cells. Each panel indicates two‐color analysis of P‐gp expression (vertical axis) and Annexin V‐binding (horizontal axis) by flow cytometry in parental and CD4‐positive populations of HALO1 cells treated with DNR (12.5 ng/mL), VCR (25 ng/mL), or Dex (250 nmol/L) for 72 hours. Cell viabilities in P‐gp‐positive and negative populations are shown at the left side of each panel. (F) Effect of P‐gp inhibitor on DNR sensitivity of t(17;19)‐ALL in vivo. NSG mice inoculated with HAOL1 cells were treated with vehicle, DNR alone, or DNR in combination with CyA for 5 days (n = 5). Vertical axis indicates survival of mice. P value between survival of mice treated with DNR alone and those treated with DNR and CyA in Kaplan‐Meier analysis is shown

To test the involvement of P‐gp in resistance to DNR and VCR in t(17;19)‐ALL cell lines, we evaluated the functional drug‐efflux activity using calcein‐AM (CAM), an ABC transporter‐dependent dye.^26^ We performed flow cytometric analyses of CAM staining in the presence or absence of ABC transporter inhibitors such as verapamil,^27^ CyA,^28^, ^29^ and nilotinib.^30^, ^31^ In HALO1 cells, CAM staining level was remarkably intensified in the presence of verapamil, CyA, or nilotinib in a dose‐dependent manner (Figure 5A). We next evaluated the effects of nilotinib on DNR and VCR sensitivity in HALO1, as well as in 697 cells [a P‐gp‐negative t(1;19)‐ALL cell line], using flow cytometry. Nilotinib alone did not induced apoptosis in either HALO1 cells or in the 697 cells (Figure 5B,C). DNR and VCR induced apoptosis in HALO1 cells more effectively in the presence of nilotinib, while sensitivities to DNR and VCR in the presence of nilotinib were unchanged in 697 cells. We further analyzed the effect of nilotinib on DNR and VCR sensitivity in two P‐gp‐positive t(17;19)‐ALL cell lines (HALO1 and UOCB1) using an alamarBlue cell viability assay. Sensitivities to DNR and VCR were significantly enhanced by nilotinib in both cell lines (Figure 5D). Sensitivity to VCR was also significantly enhanced by verapamil in both cell lines (Figure S3). To directly verify involvement of P‐gp in DNR resistance of HALO1 cells, we next established P‐gp knocked out HALO1 cells using the CRISPR/Cas9 system with a CD4 reporter.^20^ P‐gp expression was knocked out in nearly half of the CD4‐positive population (Figure S4).^20^ Then, we treated the cells with DNR (12.5 ng/mL), VCR (25 ng/mL), or Dex (250 nmol/L), for 72 hours. Two‐color analysis of P‐gp expression and Annexin V‐binding revealed that P‐gp‐negative population was sensitive to DNR and VCR (cell viabilities: 19.2% and 22.0%, respectively) whereas P‐gp‐positive population was resistant (63.2% and 66.7%, respectively). In contrast, both P‐gp‐negative and P‐gp‐positive populations were equally resistant to Dex. These in vitro observations demonstrated an involvement of P‐gp in DNR and VCR resistance in P‐gp‐positive t(17;19)‐ALL cell lines.

### 3.6 Involvement of P‐gp in daunorubicin resistance of t(17;19)‐ALL cell line in vivo

We finally tried to confirm the involvement of P‐gp in DNR resistance of t(17;19)‐ALL in vivo using NSG mice. After inoculation of HALO1 cells into NSG mice, we treated mice with DNR alone or DNR in combination with CyA for 5 days (Figure 5F). Although treatment with DNR alone did not improve survival (median survival: 34 days) in comparison with untreated control (33 days), the combination of DNR and CyA significantly improved survival (36 days, *P* = 0.018 in Kaplan‐Meier analysis).

### 3.7 Frequent RAS pathway mutations in t(17;19)‐ALL cell lines

**Figure 6:**
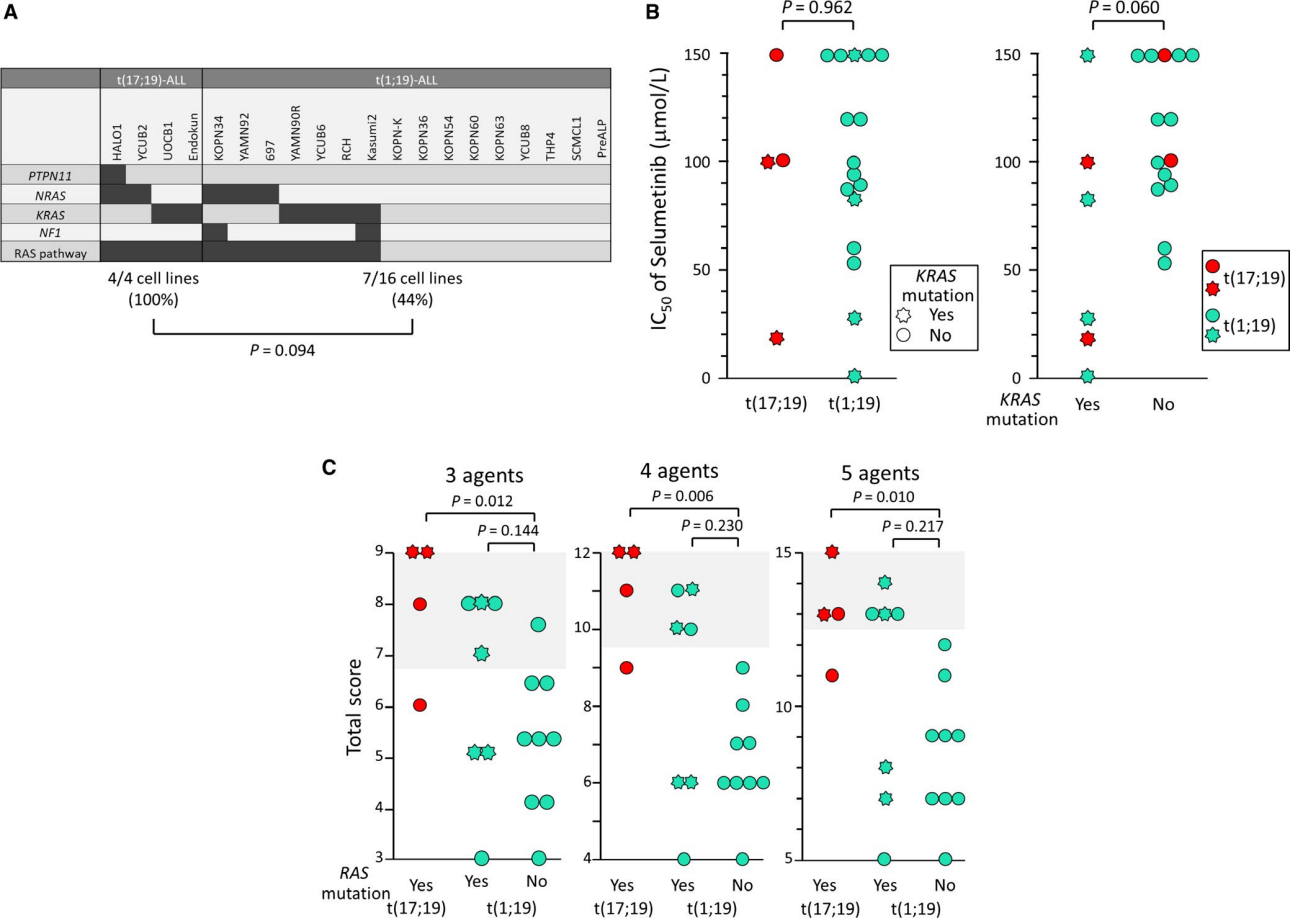
Significance of RAS pathway mutation. (A) Genetic landscape of RAS pathway in four t(17;19)‐ALL cell lines and 16 t(1;19)‐ALL cell lines. P value between incidence of mutation in t(17;19)‐ALL cell lines and that in t(1;19)‐ALL cell lines in chi‐square test is shown. (B) Sensitivity to Selumetinib. In left panel, IC50 of Selumetinib was compared between t(17;19)‐ALL cell lines and t(1;19)‐ALL cell lines. In right panel, IC50 of Selumetinib was compared between cell lines with *KRAS *mutation and those without it. Heptagrams and circles indicate cell lines with *KRAS *mutation and those without it, respectively. Red and light green symbols indicate t(17;19)‐ALL and t(1;19)‐ALL cell lines, respectively. (C) Multi‐agent resistance of t(1;19)‐ALL cell lines with RAS pathway mutation. Total score of sensitivities to three (Pred, VCR, and L‐Asp), four (Pred, VCR, L‐Asp, and DNR), and five (Pred, VCR, L‐Asp, DNR, and MAF) agents were compared among t(17;19)‐ ALL cell lines, t(1;19)‐ALL cell lines with RAS pathway mutation, and t(1;19)‐ALL cell lines without RAS pathway mutation. *P*‐values in Mann‐Whitney test are shown.

Association of gene mutation in the RAS pathway with poor therapeutic outcome in childhood ALL is controversial.^32^, ^33^, ^34^, ^35^ However, gene mutation in the RAS pathway is frequently observed in patients’ samples of t(17;19)‐ALL.^17^ Thus, we sequenced four RAS pathway genes (*KRAS*, *NRAS*, *PTPN11*, and *NF1*) in t(17;19)‐ALL and t(1;19)‐ALL cell lines using a next generation sequencer (Table 4, Figure 6A). Mutations in *PTPN11*, *NRAS*, *KRAS*, and *NF1* genes were detectable in one, two, two, and none of the four t(17;19)‐ALL cell lines, respectively, and gene mutation in RAS pathway was detectable in all t(17;19)‐ALL cell lines. In contrast, mutations in *PTPN11*, *NRAS*, *KRAS*, and *NF1* genes were detectable in none, three, four, and one of 16 t(1;19)‐ALL cell lines, respectively, and gene mutation in RAS pathway was detectable in seven out of 16 t(1;19)‐ALL cell lines. Incidence of RAS pathway mutation tended to be higher in t(17;19)‐ALL cell lines than in t(1;19)‐ALL cell lines (*P* = 0.094 in chi‐square test).

**Table 4:** RAS pathway mutation

Cell line	Gene	Allele frequency		Type of mutation	
				Codon	AA
t(17;19)					
HALO1	*NRAS*	0.831	HOM	Ggt/Agt	G12S
	*PTPN11*	0.39	HET	Tca/Cca	S502P
YCUB2	*NRAS*	0.486	HET	cAa/cTa	Q61L
Endokun	*KRAS*	0.8	HOM	ggc/gTGGgc	G13VG
UOCB1	*KRAS*	0.464	HET	Ggt/Cgt	G12R
t(1;19)					
KOPN‐K					
KOPN34	*NRAS*	0.444	HET	Ggt/Agt	G12S
	*NF1*	0.432	HET	Acc/Gcc	T940A
KOPN36					
KOPN54					
KOPN60					
KOPN63					
YAMN90R	*KRAS*	0.444	HET	gGt/gTt	G12V
YAMN92	*NRAS*	0.392	HET	gGa/gTa	G60V
YCUB6	*KRAS*	0.518	HET	gCc/gAc	A18D
YCUB8					
Kasumi2	*KRAS*	0.465	HET	Gta/Cta	V14L
	*NF1*	0.505	HET	gaG/gaT	E1699D
THP4					
SCMCL1					
697	*NRAS*	0.482	HET	gGt/gAt	G12D
RCH	*KRAS*	0.482	HET	Gta/Ata	V14I
PreALP					

### 3.8 Association between RAS pathway mutation and sensitivity to MEK inhibitor

A recent report revealed that ALL samples with *KRAS* mutation are sensitive to inhibitors of MAP kinases in vitro.^33^ Thus, we tested sensitivity of t(17;19)‐ALL cell lines to selumetinib, a MEK inhibitor that has been reported to be active against ALL with the *KRAS* mutation. We determined IC_50_ of selumetinib in t(17;19)‐ALL and t(1;19)‐ALL cell lines using an alamarBlue cell viability assay (Figure 6B). The IC_50_ of selumetinib in four t(17;19)‐ALL cell lines (two cell lines with *KRAS* mutation and two cell lines without it) (median: 100 μmol/L) was almost identical to that in 16 t(1;19)‐ALL cell lines (four cell lines with *KRAS* mutation and 12 cell lines without it) (97 μmol/L). Of note, six cell lines with *KRAS* mutation [two t(17;19)‐ALL cell lines and four t(1;19)‐ALL cell lines] were relatively more sensitive to selumetinib than 14 cell lines without *KRAS* mutation [two t(17;19)‐ALL cell lines and 12 t(1;19)‐ALL cell lines] (*P* = 0.060 in Mann‐Whitney test).

### 3.9 Relationship between RAS pathway mutation and multiagent resistance

We finally analyzed a possible association of RAS pathway mutation with drug resistance in t(17;19)‐ALL and t(1;19)‐ALL cell lines, since RAS pathway mutation was observed more frequently in t(17;19)‐ALL cell lines. Association of RAS pathway mutation with sensitivity to each of the five drugs was not statistically significant in t(1;19)‐ALL cell lines (Figure S5), but t(1;19) cell lines with RAS pathway mutation tended to be more resistant to l‐Asp than those without it (*P* = 0.050 in Mann‐Whitney test). Then, we compared the total score of three, four, and five drug sensitivities of t(1;19)‐ALL cell lines with RAS pathway mutation with those without it (Figure 6C). Although statistically insignificant, total scores of three, four, and five drug sensitivities tended to be higher in t(1;19)‐ALL cell lines with RAS pathway mutation than in those without it. Additionally, multidrug resistance to four (total score ≥ 10) and five drugs (total score ≥ 13) was significantly more common in t(1;19)‐ALL cell lines with RAS pathway mutation (four out of seven cell lines: 57.1%) than in those without it (none of nine cell lines: 0%) (*P* = 0.019 in chi‐square test).

## 4 DISCUSSION

In this study, *TCF3‐HLF* mRNA was continuously detected at high levels in a case of t(17;19)‐ALL during intensified induction therapy. Fischer et al have reported that MRD remains positive at the end of induction therapy in most t(17;19)‐ALL cases.^17^ This poor clinical response to induction therapy in t(17;19)‐ALL cases suggests that t(17;19)‐ALL is resistant to the chemotherapeutic agents used in induction therapy. Indeed, we confirmed that the IC_50_s of Pred, VCR, and DNR in four t(17;19)‐ALL cell lines were significantly higher than those in 16 t(1;19)‐ALL cell lines. We also confirmed that the combined sensitivities to three (Pred, VCR, and l‐Asp), four (Pred, VCR, l‐Asp, and DNR), and five (Pred, VCR, l‐Asp, DNR, and CPM) agents in t(17;19)‐ALL cell lines were significantly higher than in t(1;19)‐ALL cell lines. These observations suggest that resistance to multiple agents, in particular to Pred, VCR, and DNR, may be associated with poor response to induction therapy of t(17;19)‐ALL.

To verify the underlying mechanism(s) for poor response of t(17;19)‐ALL to VCR and DNR, we focused on ABC transporters,^24^, ^25^ since the IC_50_s of DNR and VCR that are sensitive to ABC transporters are correlated with each other in t(17;19)‐ALL and t(1;19)‐ALL cell lines. Consistently, among eight genes of ABC transporters, the gene expression level of *ABCB1* was significantly higher in t(17;19)‐ALL cell lines than in t(1;19)‐ALL cell lines. We also found that cell surface expression of P‐gp tended to be higher in t(17;19)‐ALL cell lines than in t(1;19)‐ALL cell lines, suggesting that P‐gp expression may be involved in resistance to VCR and DNR of P‐gp‐positive t(17;19)‐ALL cell lines. P‐gp was active in P‐gp‐positive t(17;19)‐ALL cell lines, as we found that inhibitors for P‐gp such as CyA and nilotinib intensified CAM staining and antileukemic activities of VCR and DNR. More precisely, we confirmed that knockout of P‐gp expression by CRISPR‐Cas9 overcomes resistance to VCR and DNR in a P‐gp‐positive t(17;19)‐ALL cell line. We additionally confirmed that a combination of CyA with DNR significantly prolonged survival of NSG mice inoculated with a P‐gp‐positive t(17;19)‐ALL cell line in comparison with DNR alone. Although in vivo combination activities of nilotinib and verapamil with DNR were not directly tested, these findings strongly suggest that overexpression of P‐gp is involved at least partly in resistance to VCR and DNR of P‐gp‐positive t(17;19)‐ALL cell lines. Baudis et al^36^ previously reported that *ABCB1* gene expression is detectable by RT‐PCR in Reh cells transfected with *TCF3‐HLF* under the influence of the zinc‐inducible promoter, suggesting a possibility that the *ABCB1* gene is one of the downstream target genes of *TCF3‐HLF*.

In addition to VCR and DNR, t(17;19)‐ALL cell lines were significantly more resistant to Pred than t(1;19)‐ALL cell lines. Sensitivity of ALL cells to glucocorticoids is highly associated with expression of the glucocorticoid receptor (GR). Our previous analyses demonstrated that the gene expression level of GR, analyzed by real‐time RT‐PCR of exons 8 and 9a of GR (*NR3C1*) gene (specific for *GRα* and *GRγ* isoforms), shows significant correlation with IC_50_ of Pred in 72 BCP‐ALL cell lines.^37^ Of note, GR gene expression level in t(17;19)‐ALL cell lines was almost similar to that in t(1;19)‐ALL cell lines (data not shown), suggesting that some mechanism(s) besides the GR gene expression level may be associated with resistance to Pred in t(17;19)‐ALL cell lines. Recently, P‐gp expression has been reported to be associated with resistance to glucocorticoids in inflammatory bowel disease.^38^ However, an association of higher P‐gp expression with resistance to glucocorticoids is unlikely at least in HALO1 cells, since knockout of P‐gp expression by CRISPR‐Cas9 did not overcome Dex resistance.

A previous report revealed that genes in RAS pathway are frequently mutated in clinical samples of t(17;19)‐ALL cases but not in t(1;19)‐ALL cases.^17^ In the present study, RAS pathway mutation was detected relatively more frequently in t(17;19)‐ALL cell lines than in t(1;19)‐ALL cell lines; all four t(17;19)‐ALL cell lines and seven of the 16 t(1;19)‐ALL cell lines had mutations. Thus, RAS pathway mutation seems to be more frequent in the cell lines than in the clinical samples, suggesting that RAS pathway mutation may be advantageous for in vitro cell growth and/or cell survival of the cell lines. Of note, two t(17;19)‐ALL cell lines and four t(1;19)‐ALL cell lines with the *KRAS* mutation were relatively more sensitive to selumetinib, a MEK inhibitor, than two t(17;19)‐ALL cell lines and 12 t(1;19)‐ALL cell lines without the mutation. This higher sensitivity to selumetinib seems to be consistent with the above hypothesis that RAS pathway mutation may provide an advantage for in vitro cell growth and/or cell survival of the cell lines. Furthermore, among 16 t(1;19)‐ALL cell lines, multidrug resistance was significantly more common in the cell lines with RAS pathway mutation than those without it. These observations suggest that frequent RAS pathway mutation may be involved at least partly in the aggressive clinical course of t(17;19)‐ALL.

In summary, our observations of a large panel of cell lines revealed that t(17;19)‐ALL cell lines were significantly more resistant to multiple agents in induction therapy in comparison with t(1;19)‐ALL cell lines. Although there are some limitations in using the cell lines in drug sensitivity studies, our findings seem to be consistent with the clinical notion that t(17;19)‐ALL is resistant to intensified induction therapy in comparison with t(1;19)‐ALL. Thus, these cell lines may be optional tools to study the mechanism(s) for drug resistance and to verify the activities of newly developed compounds in t(17;19)‐ALL.

## CONFLICT OF INTEREST

The authors declare no competing financial interests.

## AUTHORSHIP

AW performed the research, analyzed the data, and wrote the paper; T. Inukai designed the research study, performed the research, analyzed the data, and wrote the paper as a principal investigator; KK, MA, MT, TF, HF, TN, KT, T. Ito, TT, DH, TS, SS, HO, KA, and K.G performed the research; JF, HG, ME, TL, MK, MM, JK T.Inaba, and HT contributed essential samples and cell lines; EI, AO, and KS supervised the project; and all authors contributed to the final draft.
