# Supplementary figures and images for: Resistance of t(17;19)‐acute lymphoblastic leukemia cell lines to multiagents in induction therapy
Source: Cancer Med. 2019 Jul 15;8(11):5274–88. doi: 10.1002/cam4.2356 (PMC6718581; doi:10.1002/cam4.2356)

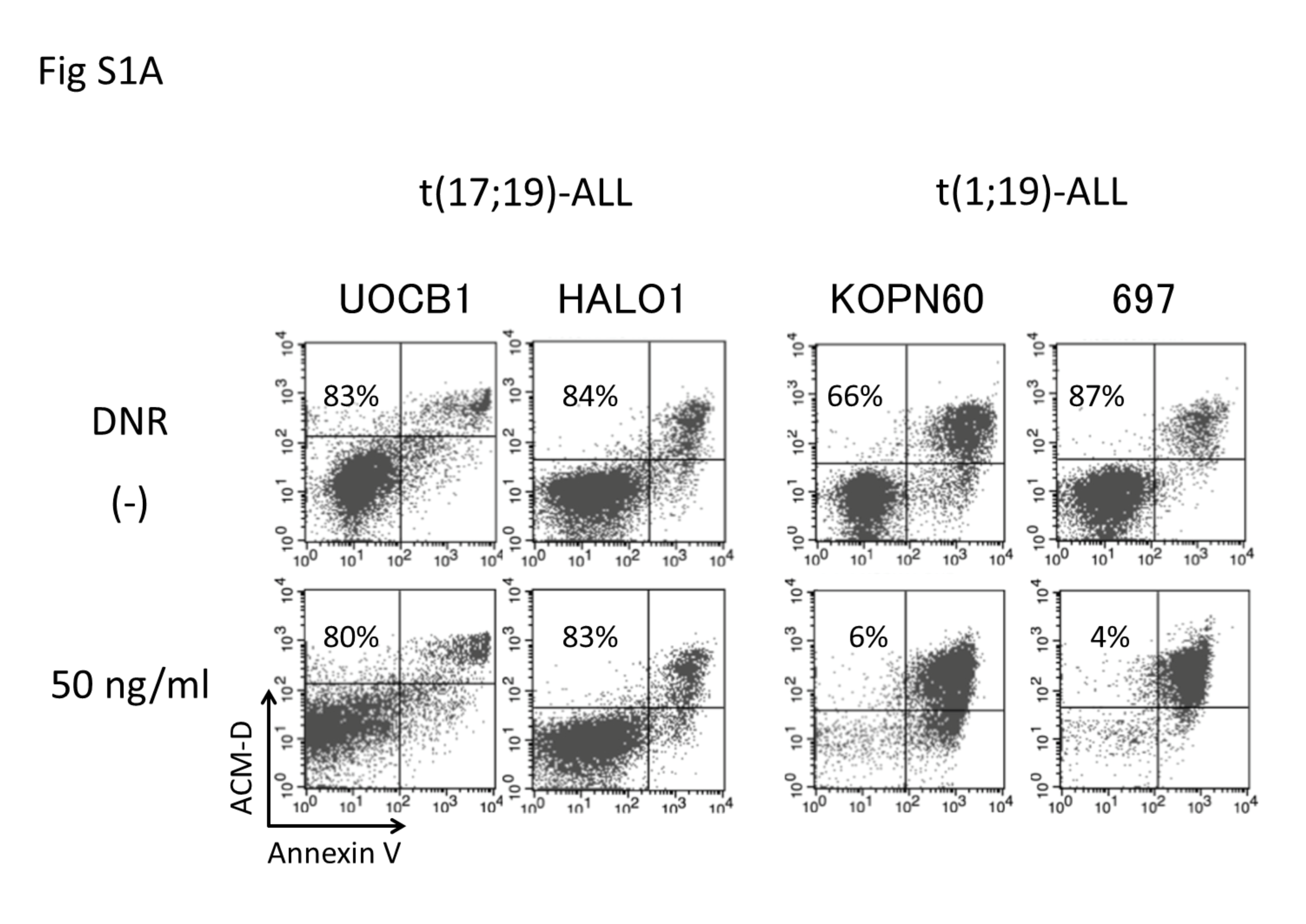

Supplement: Supplementary file 1 [file CAM4-8-5274-s001.tif]

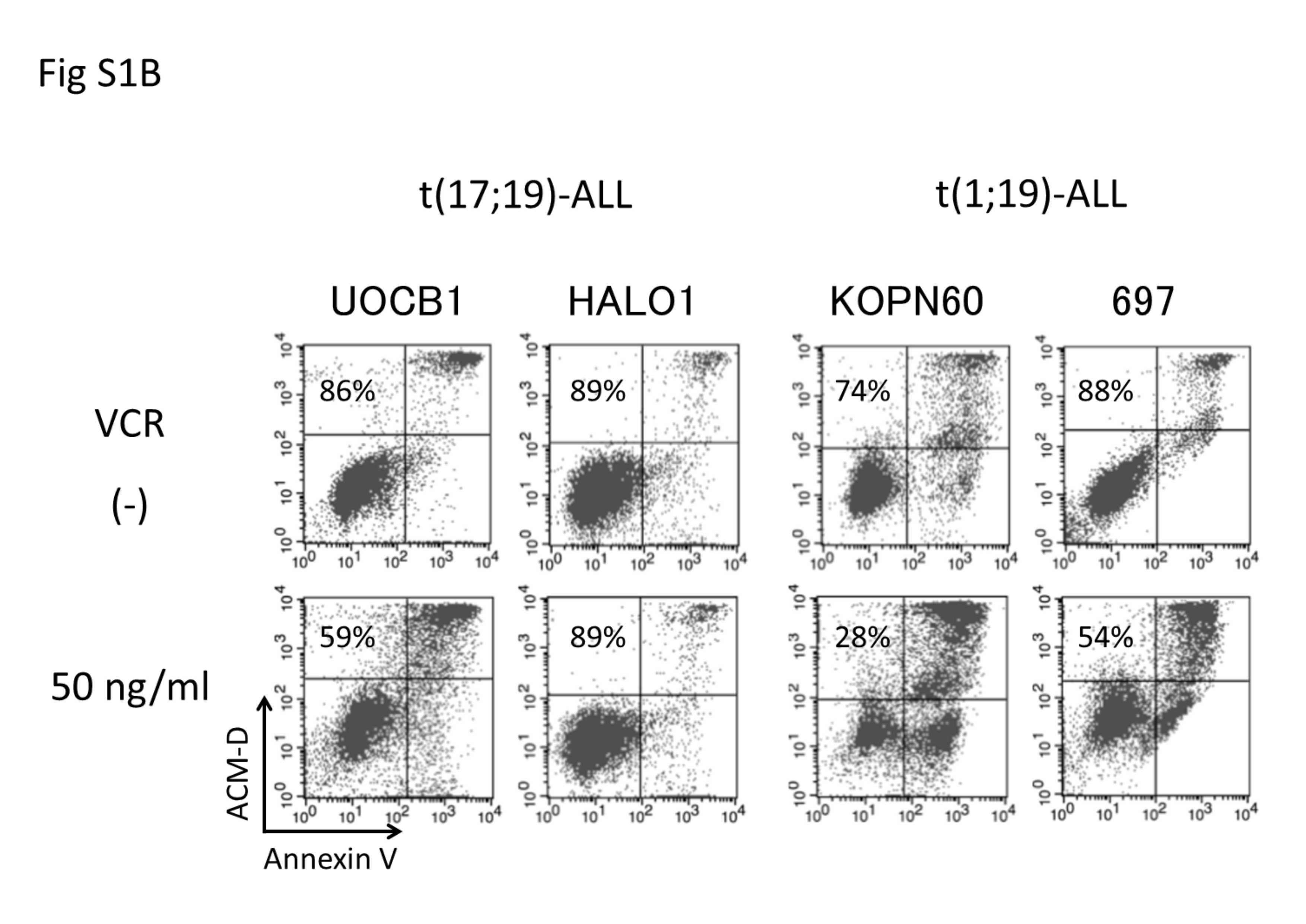

Supplement: Supplementary file 2 [file CAM4-8-5274-s002.tif]

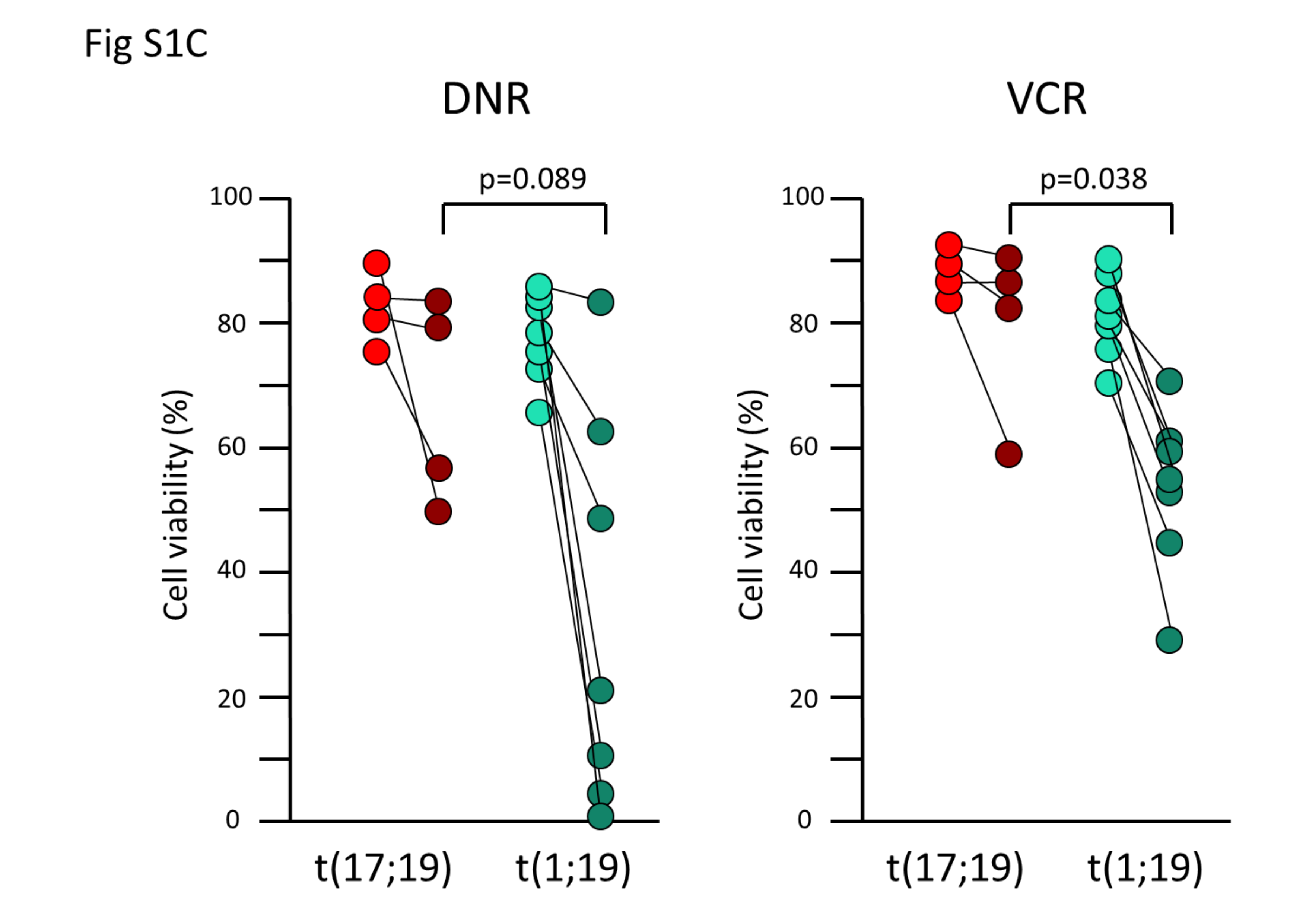

Supplement: Supplementary file 3 [file CAM4-8-5274-s003.tif]

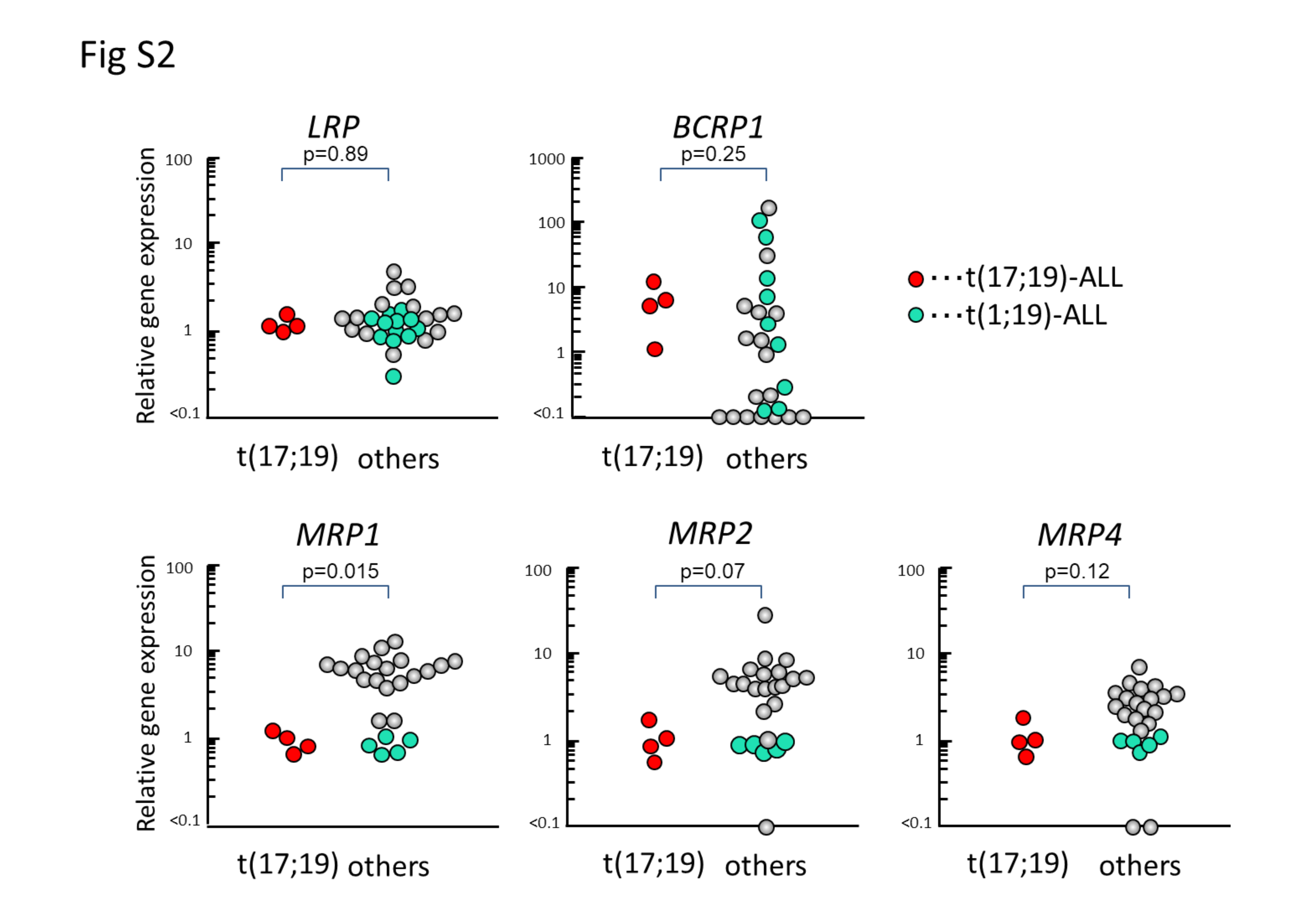

Supplement: Supplementary file 4 [file CAM4-8-5274-s004.tif]

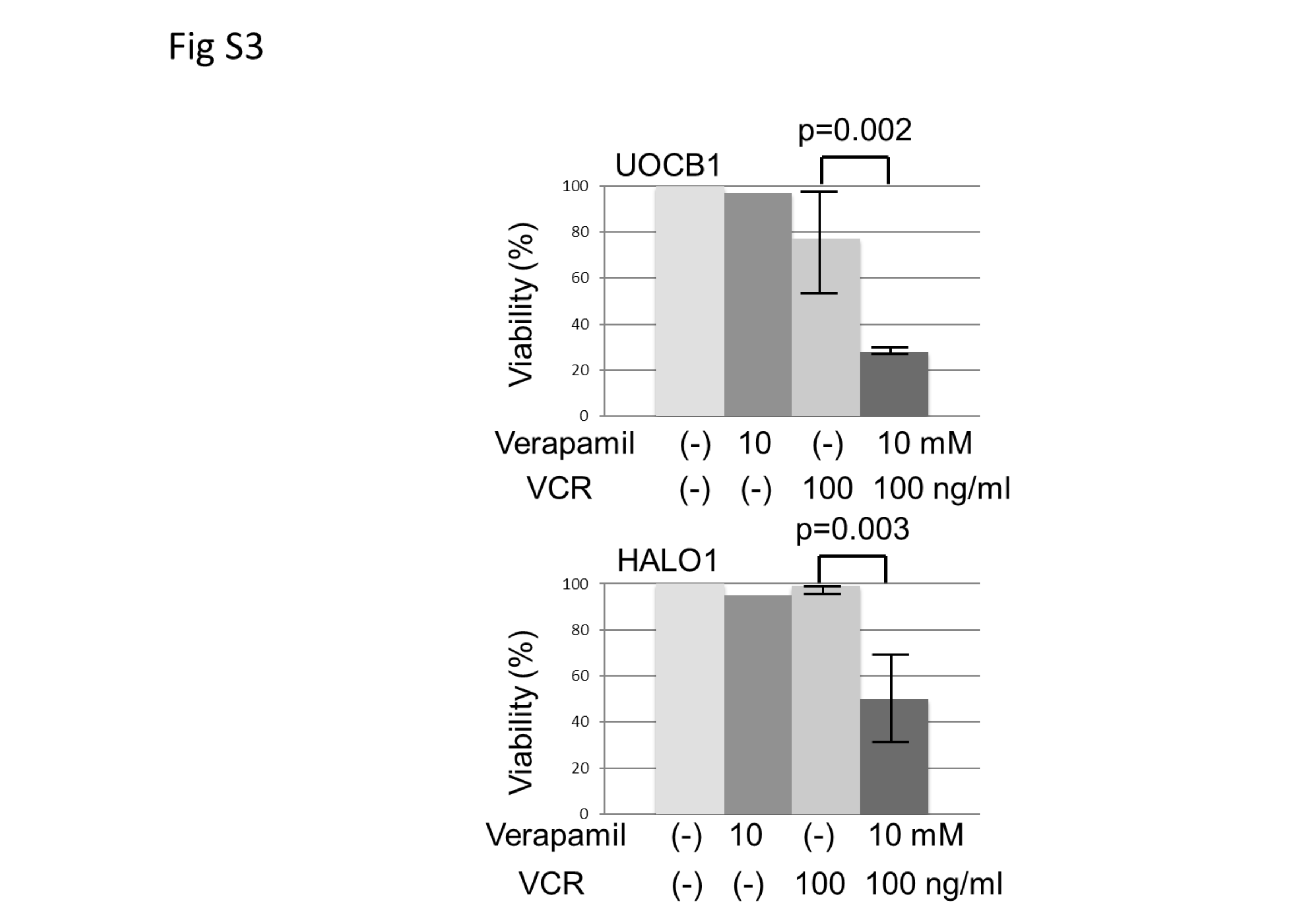

Supplement: Supplementary file 5 [file CAM4-8-5274-s005.tif]

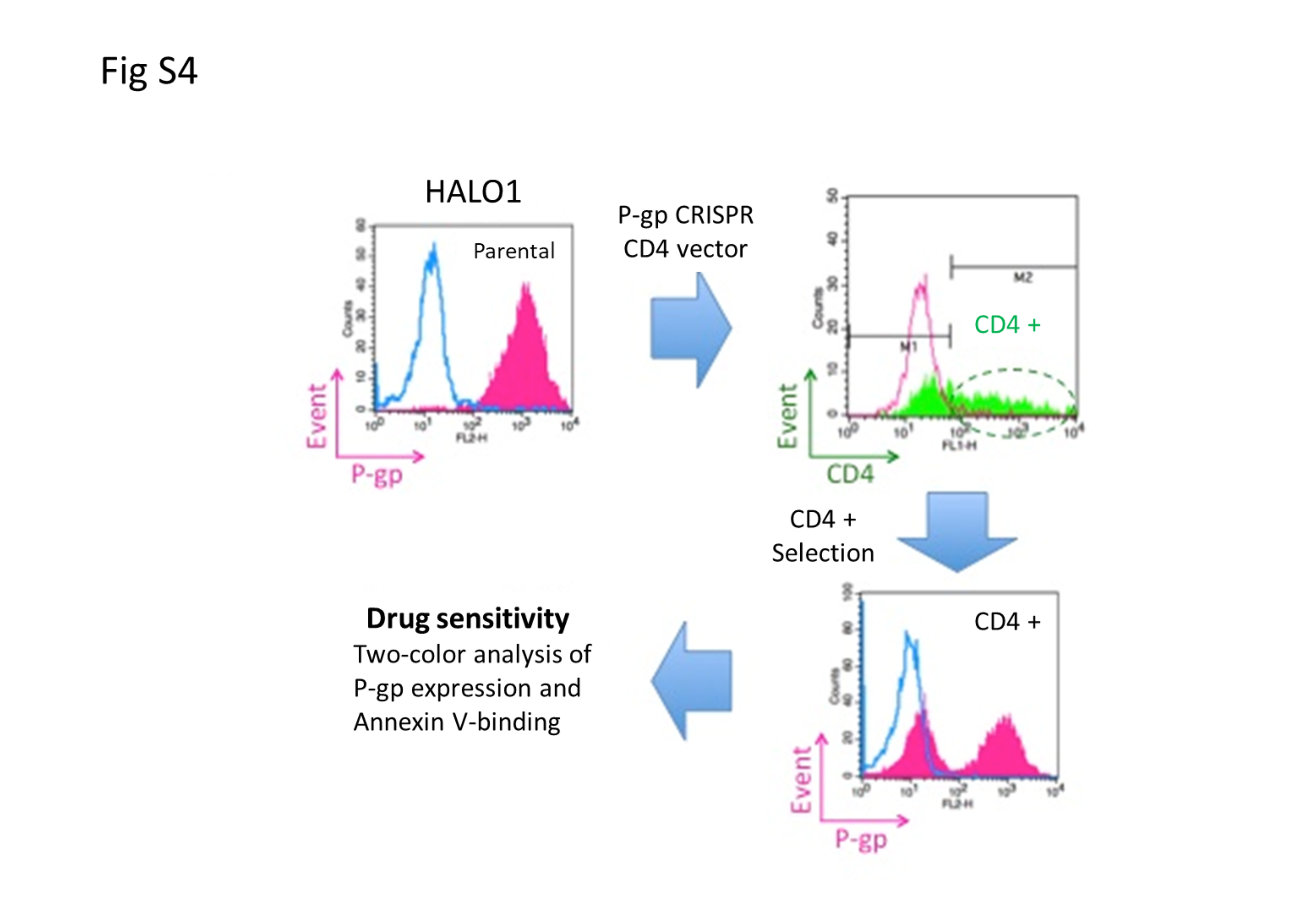

Supplement: Supplementary file 6 [file CAM4-8-5274-s006.tif]

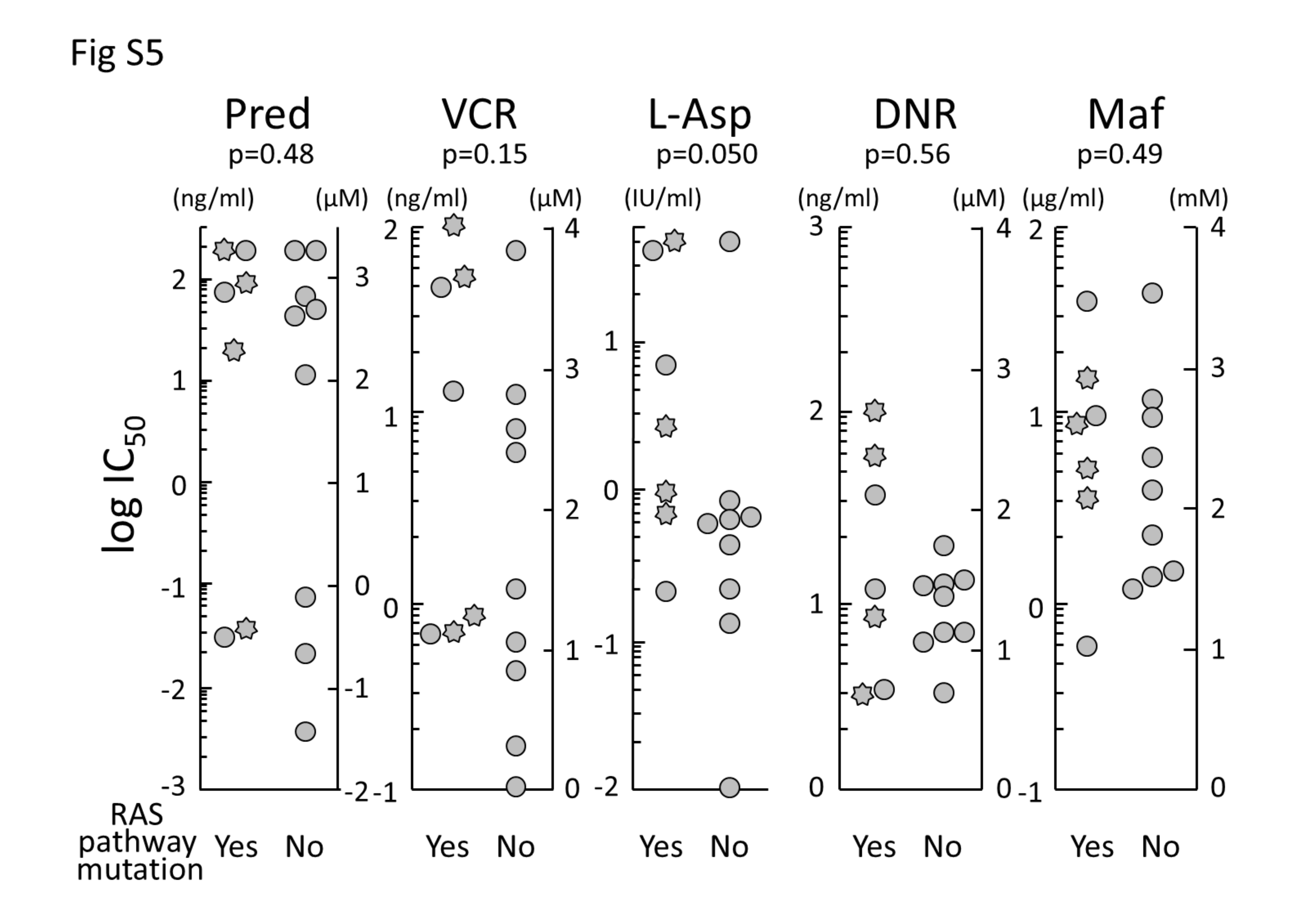

Supplement: Supplementary file 7 [file CAM4-8-5274-s007.tif]
