# Supplementary material for: Resistance of t(17;19)‐acute lymphoblastic leukemia cell lines to multiagents in induction therapy
Source: Cancer Med. 2019 Jul 15;8(11):5274–88. doi: 10.1002/cam4.2356 (PMC6718581; doi:10.1002/cam4.2356)
Supplement: Supplementary file 8 [file CAM4-8-5274-s008.docx]

| Table S1. List of cell lines | | |  |  |  |  |  |  |
| --- | --- | --- | --- | --- | --- | --- | --- | --- |
| Type | Cell line | Age | Sex |  | Type | Cell line | Age | Sex |
| t(17;19) |  |  |  |  | *MLL*+ |  |  |  |
|  | HALO1 | 17 | F |  |  | KOPN-1^*^ | <1 | F |
|  | YCUB2 | 4 | M |  |  | KOCL-33 | <1 | F |
|  | Endokun^*^ | 14 | F |  |  | KOCL-44^*^ | 2 | F |
|  | UOCB1 | 15 | F |  |  | KOCL-45^*^ | 1 | M |
|  |  |  |  |  |  | KOCL-50^*^ | 1 | F |
| t(1;19) |  |  |  |  |  | KOCL-58 | <1 | M |
|  | KOPN-K^*^ | 5 | M |  |  | KOCL-69 | <1 | F |
|  | KOPN34 | 61 | M |  |  |  |  |  |
|  | KOPN36^*^ | 3 | F |  | Ph+ |  |  |  |
|  | KOPN54^*^ | 9 | M |  |  | KOPN-30bi | 8 | M |
|  | KOPN60 | 14 | M |  |  | KOPN-57bi | 11 | M |
|  | KOPN63^*^ | 12 | F |  |  | KOPN-66bi^*^ | 10 | M |
|  | YAMN90R* | 4 | M |  |  | KOPN-72bi^*^ | 9 | M |
|  | YAMN92* | 14 | M |  |  | YAMN-73^*^ | 6 | F |
|  | YCUB6 | 12 | M |  |  | SU-Ph2 | 50 | F |
|  | YCUB8 | 13 | F |  |  |  |  |  |
|  | Kasumi2 | 15 | M |  | Others |  |  |  |
|  | THP4 | 2 | M |  |  | KOPN-32^*^ | 2 | F |
|  | SCMC-L1 | <1 | F |  |  | KOPN-35^*^ | 2 | F |
|  | 697 | 12 | M |  |  | KOPN-41 | 4 | M |
|  | RCH | 8 | F |  |  | KOPN-62 | 72 | M |
|  | PreALP | 6 | F |  |  | KOPN-70^*^ | 10 | F |
|  |  |  |  |  |  | KOPN-79^*^ | 13 | F |
|  |  |  |  |  |  | Reh | 15 | F |
|  |  |  |  |  |  | Nalm6 | 19 | M |
| *established at relapse | |  |  |  |  |  |  |  |
| F, female; M, male. | |  |  |  |  |  |  |  |

| Table S2. List of drugs | |  |
| --- | --- | --- |
| Drug | Company |  |
| Dexamethasone | Sigma-Aldrich, MO, USA |  |
| (Dex) |  |  |
| Prednisolone | Sigma-Aldrich, MO, USA |  |
| (Pred) |  |  |
| Vincristine | Cayman chemical, MI, USA |  |
| (VCR) |  |  |
| Daunorubicin | Cayman chemical, MI, USA |  |
| (DNR) |  |  |
| L-asparaginase | Kyowa Hakko Kirin, Tokyo, Japan |  |
| (L-Asp) |  |  |
| Mafosfamide | Toronto Research Chemicals, ON, Canada |  |
| (Maf) |  |  |
| Cyclosporine A | Sigma-Aldrich, MO, USA |  |
| (CyA) |  |  |
| Selumetinib | LC Laboratories, MA, USA |  |
|  |  |  |
| Verapamil | Focus Biomolecules, PA, USA |  |
|  |  |  |
| Nilotinib | AdipoGen Life Sciences, Liestal, Switzerland |  |
|  |  |  |
